# Laser microdissection-based microproteomics of the hippocampus of a rat epilepsy model reveals regional differences in protein abundances

**DOI:** 10.1038/s41598-020-61401-8

**Published:** 2020-03-10

**Authors:** Amanda M. do Canto, André S. Vieira, Alexandre H.B. Matos, Benilton S. Carvalho, Barbara Henning, Braxton A. Norwood, Sebastian Bauer, Felix Rosenow, Rovilson Gilioli, Fernando Cendes, Iscia Lopes-Cendes

**Affiliations:** 1 Department of Medical Genetics and Genomic Medicine, School of Medical Sciences, University of Campinas (UNICAMP), Campinas, Brazil; 2 Department of Functional and Structural Biology, Biology Institute, University of Campinas (UNICAMP), Campinas, Brazil; 3 Department of Statistics, Institute of Mathematics, Statistics and Scientific Computing, University of Campinas, (UNICAMP), Campinas, Brazil; 4 Expesicor Inc, Kalispell, MT USA; 5 Department of Neurology, Epilepsy Centre Hessen, Philipps-University Marburg, Marburg, Germany; 6 Epilepsy Centre Frankfurt Rhine-Main, Goethe-University Frankfurt, Frankfurt/Main, Frankfurt, Germany; 7 Laboratory of Animal Quality Control, University of Campinas (UNICAMP), Campinas, Brazil; 8 Department of Neurology, School of Medical Sciences, University of Campinas (UNICAMP), Campinas, Brazil; 9 Brazilian Institute of Neuroscience and Neurotechnology (BRAINN), Campinas, Brazil

**Keywords:** Epilepsy, Molecular neuroscience

## Abstract

Mesial temporal lobe epilepsy (MTLE) is a chronic neurological disorder affecting almost 40% of adult patients with epilepsy. Hippocampal sclerosis (HS) is a common histopathological abnormality found in patients with MTLE. HS is characterised by extensive neuronal loss in different hippocampus sub-regions. In this study, we used laser microdissection-based microproteomics to determine the protein abundances in different regions and layers of the hippocampus dentate gyrus (DG) in an electric stimulation rodent model which displays classical HS damage similar to that found in patients with MTLE. Our results indicate that there are differences in the proteomic profiles of different layers (granule cell and molecular), as well as different regions, of the DG (ventral and dorsal). We have identified new signalling pathways and proteins present in specific layers and regions of the DG, such as PARK7, RACK1, and connexin 31/gap junction. We also found two major signalling pathways that are common to all layers and regions: inflammation and energy metabolism. Finally, our results highlight the utility of high-throughput microproteomics and spatial-limited isolation of tissues in the study of complex disorders to fully appreciate the large biological heterogeneity present in different cell populations within the central nervous system.

## Introduction

Currently, it is well recognised that proteins and their biological roles are multifaceted and complex since all biological mechanisms rely on correct protein function. However, a major part of protein cellular machinery remains unknown, especially in disease-related processes. Therefore, studying proteins, specifically their presence and abundance, is relevant when addressing complex mechanisms leading to disease^1^. Laser microdissection-based microproteomics allows for the study of the proteome of enriched specific cell types of interest obtained from a small amount of tissue (100 μg or less)^1–3^.

Epilepsy is a chronic neurological disorder affecting around 2% of the world population^27/02/2020 18:22:00^. It presents a wide variety of clinic manifestations, aetiologies, severities, and prognoses. However, the occurrence of an epileptic seizure, caused by abnormal neuronal discharges, is the common feature of all types of epilepsy^4^. Mesial temporal lobe epilepsy (MTLE) is the most frequent form in adults, present in 40% of patients with epilepsy, many of whom do not respond to clinical treatment^5–8^.

The most frequent histopathological characteristic found in MTLE is hippocampal sclerosis (HS)^9,10^, which can be divided into three types according to the International League Against Epilepsy (ILAE) classification. Type 1 is the most common, and it is characterised by neuronal loss that mainly affects the CA1 and CA3 regions (*Cornu Ammonis*) of the hippocampus^11–14^. The hippocampus is a medial temporal lobe structure that is involved in cognitive functions, such as recent memory formation and spatial navigation. The dentate gyrus (DG) is an integral part of the hippocampal formation and may be subdivided into dorsal and ventral regions in a longitudinal disposition in rodents and posterior and anterior regions in humans^15–20^. Despite their similar neuronal compositions, the dorsal and ventral portions of the DG have different connections with cortical and subcortical areas, which indicate a variation in the structure and function throughout the dorsal-ventral axis^21^. Furthermore, the DG has three layers: the molecular layer (ML) is formed mostly by dendrites of granule cells and axons from the perforant pathway, the granule cell layer (GL) is comprised of granule cell bodies, and the polymorphic layer contains a number of different cell types^22,23^. Considering these characteristics of the hippocampus and that it is the most affected structure in patients with MTLE due to the HS, the precise isolation of different cell populations and cell compartments, such as the DG, may improve the specificity of the information obtained using high-throughput techniques, such as microproteomics; thus, leading to the identification of unique mechanisms that take place in precise anatomic regions and cell types^22^.

In this study, for the first time, we present the proteomic profile from different regions and layers of the DG, obtained by laser microdissection-based microproteomics of an epilepsy rodent model displaying a HS phenotype similar to the classical observed in patients with MTLE.

## Results

Electrical stimulation was performed one week after electrode placement. First, the animals were stimulated for 30 minutes on 2 consecutive days and for 8 hours on the third day^24^. Animals were euthanised 15 days after the last stimulation session, and brains were collected and processed for laser microdissection. The time point of 15 days for the collection of tissue was chosen because it is part of the ‘silent phase’ of the model as described previously^24^. At this time point, an extensive neuronal loss is observed, especially at the CA1, CA2, and CA3 regions of the hippocampus of stimulated rats, which likely occurs due to the electrical stimulation. Therefore, the 15-day time-point fairly reflects the molecular and cellular mechanisms that underly the epileptogenesis process that occurs in the perforant pathway stimulation (PPS) model. We have used the term ‘epileptogenesis’ in the present work, according to the new terminology revised by the ILAE^25^.

**Figure 1:**
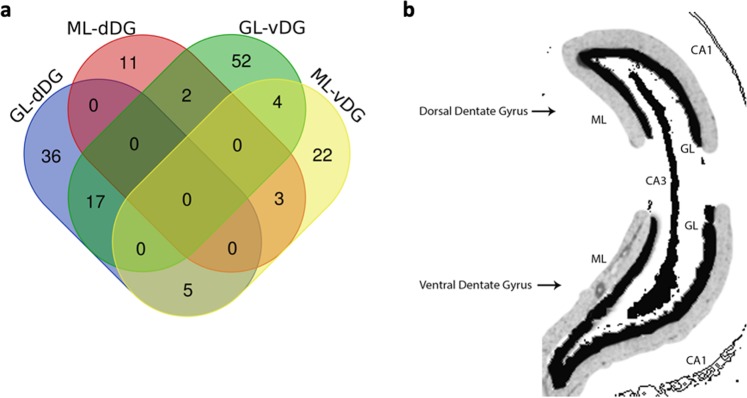
Venn diagram representing the abundant proteins identified in each region from the dentate gyrus (DG) of the perforant pathway stimulation (PPS) model and a representative image from the DG. (**a**) Venn diagram. The blue circle represents the proteins identified at the granule cell layer of the dorsal dentate gyrus (GL-dDG) and the red circle represents the proteins identified at the molecular layer of the dorsal dentate gyrus (ML-dDG), highlighting that no protein was common between the two layers of the dorsal DG. The yellow circle represents the proteins identified in the molecular layer of the ventral dentate gyrus (ML-vDG), and the green circle represents the proteins identified in the granule cell layer of the ventral dentate gyrus (GL-vDG). (**b**) Schematics of the DG, indicating how the DG is divided into dorsal and ventral subfields, as well as how the two layers, the granule cell layer (GL) in black and the molecular layer (ML) in grey, are divided.

Overall, we found that more than 50% of the proteins differentially expressed at p < 0.05 were uniquely expressed in specific layers and regions of the hippocampus. Specifically, there were 36 out of 58 proteins expressed solely in the granule cell layer of the dorsal dentate gyrus (GL-dDG), 11 out of 16 in the molecular layer of the dorsal dentate gyrus (ML-dDG), 50 out of 75 in the granule cell layer of the ventral dentate gyrus (GL-vDG), and 21 out of 34 in the molecular layer of the ventral dentate gyrus (ML-vDG) (Fig. 1).

**Figure 2:**
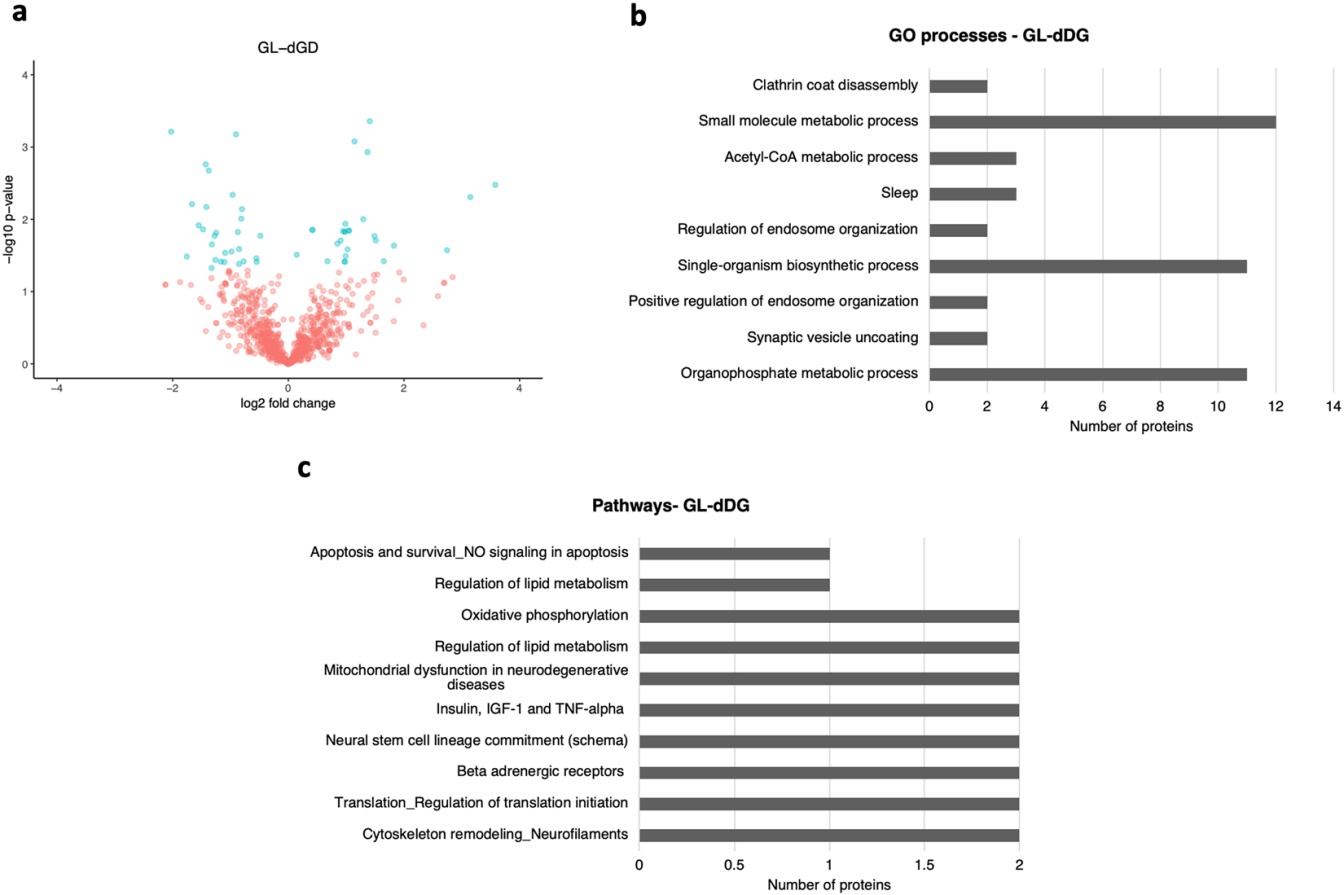
Differentially abundant proteins from the GL-dDG. (**a**) Volcano plot showing the differentially abundant proteins identified at the GL-dDG from the hippocampus of the PPS model comparing stimulated animals with sham controls. (**b**) The main enriched GO processes identified considering the most represented proteins in the GL-dDG of stimulated animals. (**c**) The main enriched pathways identified considering the most represented proteins identified in the GL-dDG.

In the GL-dDG, we identified a total of 959 proteins. Comparing the GL-dDG of epileptic rats with sham control rats we found a differential abundance of 58 proteins (at a significance level of 5%), of which 32 were downregulated and 26 were upregulated (Fig. 2a; for a complete list of proteins, refer to Supplementary Dataset 1). The most significant (p-value <0.05) gene ontology (GO) processes among the differentially abundant proteins were organophosphate metabolic processes, synaptic vesicle uncoating, positive regulation of endosome organisation, and single-organism biosynthetic processes (Fig. 2b; for a complete list, refer to Supplementary Dataset 2). Furthermore, the main enriched pathways were cytoskeleton remodelling (neurofilaments), translation (regulation of translation initiation), development (beta-adrenergic receptors in brown adipocyte differentiation), and development (neural stem cell lineage commitment), as shown in Fig. 2c (for a complete list refer to Supplementary Dataset 3).

**Figure 3:**
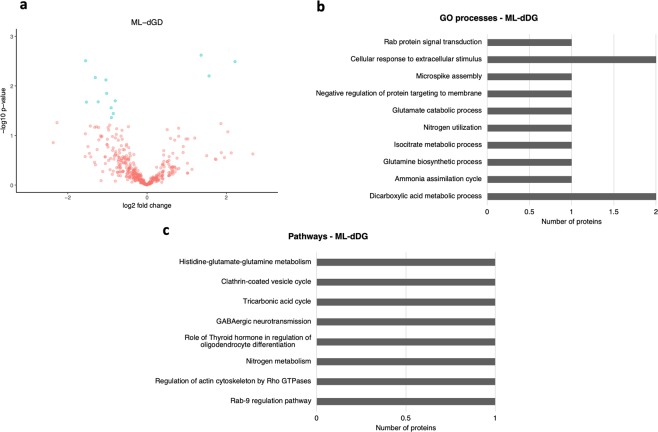
Differentially abundant proteins identified in the ML-dDG. (**a**) Volcano plot showing the differentially abundant proteins identified in the ML-dDG from the hippocampus of the PPS model comparing stimulated animals with sham controls. (**b**) The main enriched GO processes identified considering the most represented proteins in the ML-dDG of stimulated animals. (**c**) The main enriched pathways identified considering the most represented proteins identified in the ML-dDG.

When comparing the ML-dDG of epileptic rats with sham controls, we identified a total of 448 proteins. We found that 16 of them were differentially expressed (p < 0.05), where 11 were downregulated and 5 upregulated (Fig. 3a; for a complete list of proteins, refer to Supplementary Dataset 4). The most significant (p < 0.05) GO processes involving these 16 proteins included the dicarboxylic acid metabolic process, the ammonia assimilation cycle, the glutamine biosynthetic process, and the isocitrate metabolic process (Fig. 3b; for a complete list refer to Supplementary Dataset 5). Moreover, the main enriched pathways were transport (Rab-9 regulation pathway), cytoskeleton remodelling (regulation of actin cytoskeleton by Rho GTPases), nitrogen metabolism, and development (role of thyroid hormone in the regulation of oligodendrocyte differentiation) as shown in Fig. 3c (for a complete list refer to Supplementary Dataset 6).

**Figure 4:**
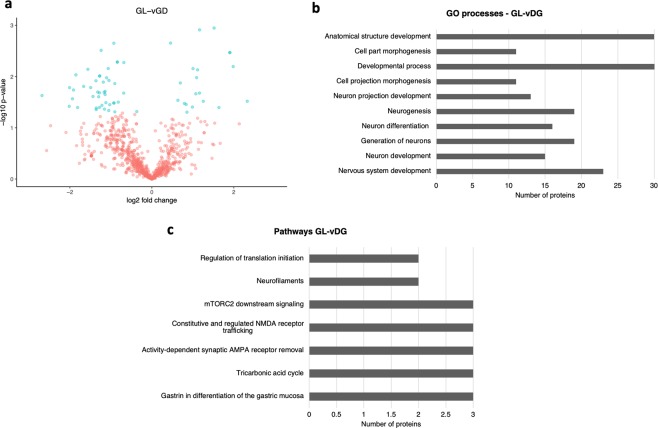
Differentially abundant proteins found in the GL-vDG. (**a**) Volcano plot showing the differentially abundant proteins identified in the GL-vDG from the hippocampus of the PPS model comparing stimulated animals with sham controls. (**b**) The main enriched GO processes identified considering the most represented proteins in the GL-vDG of stimulated animals. (**c**) The main enriched pathways identified considering the most represented proteins identified in the GL-vDG.

In the comparison of the GL-vDG from the epileptic rats with sham controls we identified 898 proteins, of which, 75 were differentially expressed (p < 0.05). In total, 52 were downregulated and 23 were upregulated, as shown in Fig. 4a (for a complete list of proteins refer to Supplementary Dataset 7). The most significant GO processes identified (p < 0.05) were nervous system development, neuron development, generation of neurons, and neuron differentiation (Fig. 4b; for a complete list refer to Supplementary Dataset 8). The main enriched pathways were development (gastrin in the differentiation of the gastric mucosa), tricarbonic acid cycle, neurophysiological process (activity-dependent synaptic AMPA receptor removal), neurophysiological process (constitutive and regulated NMDA receptor trafficking as shown in Fig. 4c (for a complete list refer to Supplementary Dataset 9).

**Figure 5:**
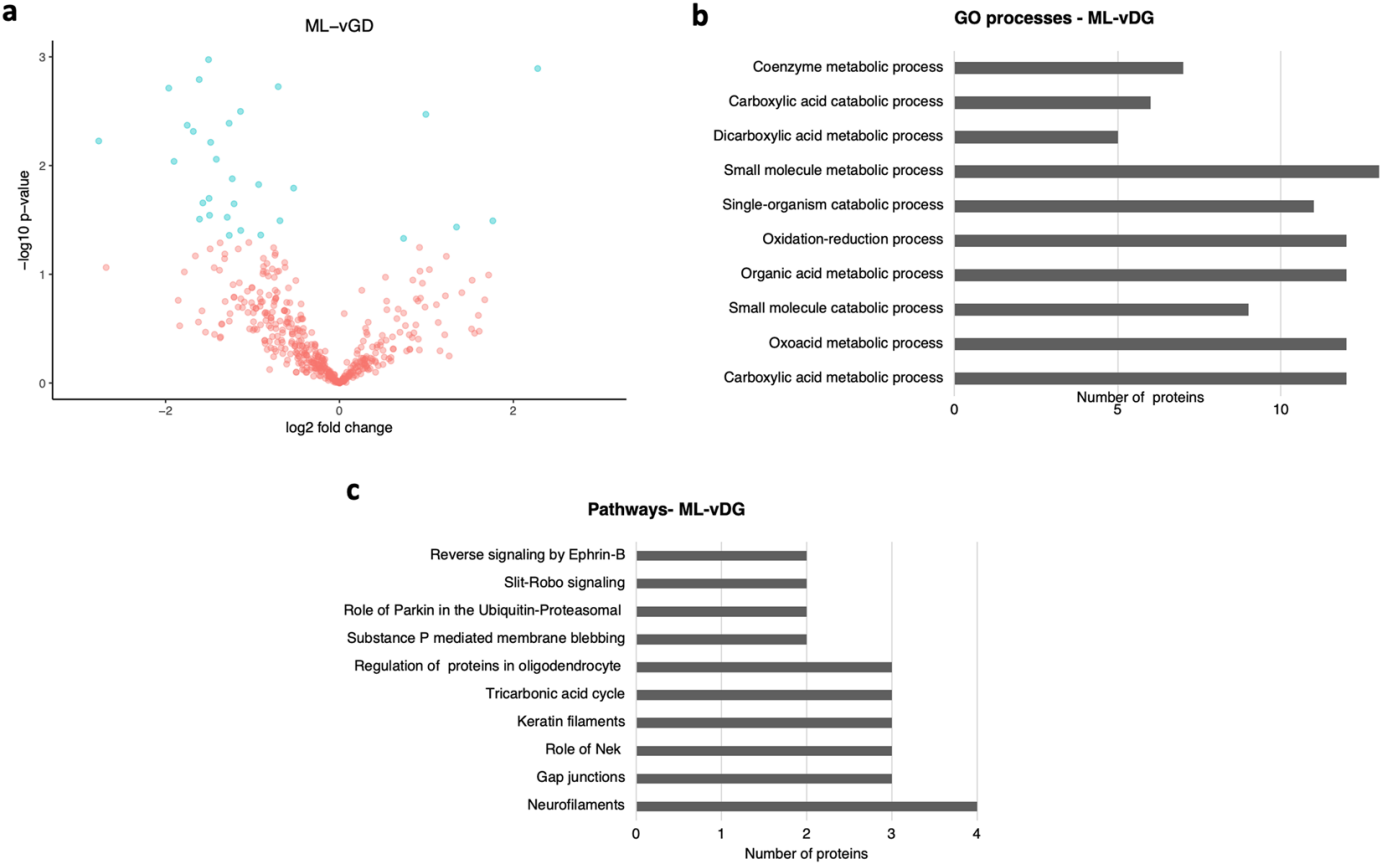
Differentially abundant proteins identifed in the ML-vDG. (**a**) Volcano plot showing the differentially abundant proteins identified in the ML-vDG from the hippocampus of the PPS model comparing stimulated animals with sham controls. (**b**) The main enriched GO processes identified considering the most represented proteins in the ML-vDG of stimulated animals. (**c**) The main enriched pathways identified considering the most represented proteins identified in the ML-vDG.

In the final comparison of the ML-vDG of epileptic rats with sham controls, we identified 534 proteins, of which, 34 were differentially expressed at p < 0.05. Of these, 29 were downregulated and 5 were upregulated (Fig. 5a; for a complete list refer to Supplementary Dataset 10). The most significant GO processes identified were carboxylic acid metabolic process, oxoacid metabolic process, the small molecule catabolic process, and organic acid metabolic process (Fig. 5b; for a complete list refer to Supplementary Dataset 11). The most significantly enriched pathways were cytoskeleton remodelling: (neurofilaments), cell adhesion (gap junctions), cell cycle (role of Nek in cell cycle regulation), and cytoskeleton remodelling (keratin filaments) as shown in Fig. 5c (for a complete list refer to Supplementary Dataset 12).

Overall, there were two main pathways present in all layers and regions of the DG: inflammation and energy metabolism. We identified the following inflammatory pathways in the GL-dDG: role of integrins in NK cells cytotoxicity, PD-1-induced metabolic changes in T cells, antigen presentation by MHC class I, classical pathway and the effect of INDO on T cell metabolism. The enriched inflammatory pathways in the GL-vDG were CRTH2 signalling in Th2 cells, MIF (the neuroendocrine-macrophage connector), function of MEF2 in T lymphocytes, IL-16 signalling pathway, reactive oxygen species (ROS) in IL-4 signalling, IL-3 signalling via ERK and PI3K, regulatory role of C1q in platelet activation, MIF-JAB1 signalling, and NETosis in SLE. In contrast, the ML-dDG did not present any enriched inflammatory pathways; and the ML-vDG presented the T cell receptor signalling pathway. In addition, we found the following energy metabolism pathways: oxidative phosphorylation (in the GL-dDG), glycolysis, gluconeogenesis (in the GL-dDG and ML-vDG), glycogen metabolism (in the ML-vDG), pyruvate metabolism (in the GL-dDG), tricarbonic acid cycle (in the GL-vDG, ML-vDG, and ML-dDG), and mitochondrial dysfunction in neurodegenerative diseases (in the GL-dDG, GL-vDG, and ML-vDG).

**Figure 6:**
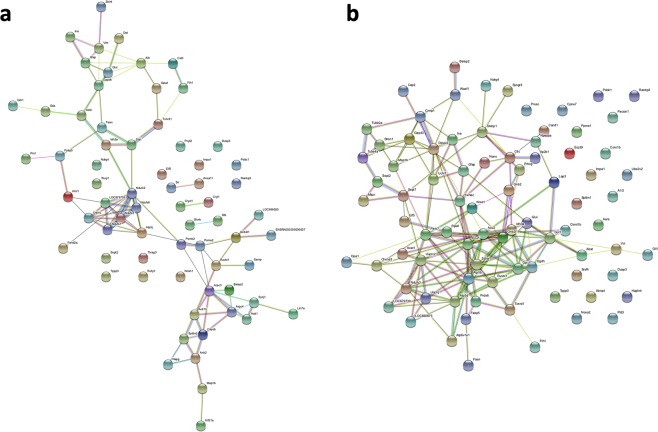
Representation of the STRING protein–protein interaction (PPI) network. The PPI networks were obtained using the STRING database with the differentially expressed proteins from the dorsal and ventral DG. (**a**) Dorsal DG PPI network. (**b**) Ventral DG PPI network.

Remarkably, we found 49 proteins that have never been reported as possibly being involved in epilepsy in an animal model or human tissue. These proteins are listed in Table 1. Furthermore, we performed a protein–protein interaction (PPI) analysis using the STRING database. For both dorsal and ventral DG, the proteins identified revealed more interactions among themselves than what would be expected for a random set of proteins. Such an enrichment indicates that the proteins are at least partially biologically connected as a group. For the dorsal DG, we obtained 63 nodes and 65 edges. The expected number of edges was 34, and the PPI enrichment p-value was 1 × 10^−6^. For the ventral DG, the data showed 82 nodes and 167 edges, while the expected number of edges was 49 and the PPI enrichment p-value was 1 × 10^−16^. For the reactome pathways in the dorsal DG, we found two enriched pathways: metabolism and membrane trafficking. While for the ventral DG, we found 24 enriched pathways, the most significant were L1CAM interactions, transmission across chemical synapses, recycling pathway of L1, and axon guidance. The protein networks are shown in Fig. 6.

**Table 1:** Proteins found to be differentially expressed in the present study, which have never been reported as associated with epilepsy.

Protein name	Accession number	Gene symbol	Region
Uncharacterised protein	A0A0G2K613	A0A0G2K613	GL-dDG
AP2-associated protein kinase 1	F1LRI7	Aak1	GL-dDG
ABRA C-terminal-like	D3ZSL2	Abracl	ML-vDG
ARP1 actin-related protein 1 homolog B	B2RYJ7	Actr1b	GL-dDG
Actin-related protein 2/3 complex subunit 3	B2GV73	Arpc3	ML-dDG
ATP synthase-coupling factor 6, mitochondrial	P21571	Atp5j	GL-dDG
ATPase, H + transporting, V1 subunit E isoform 1, isoform CRA_a	G3V7L8	Atp6v1e1	GL-vDG
ATPase H + -transporting V1 subunit H	A0A0G2K9J2	Atp6v1h	GL-vDG
F-actin-capping protein subunit beta	Q5XI32	Capzb	GL-dDG
MICOS complex subunit	D3ZUX5	Chchd3	GL-vDG
Copine VII (Predicted), isoform CRA_b	D3ZWR4	Cpne7	ML-vDG
Crystallin zeta-like 1	Q5XI39	Cryzl1	GL-dDG
Divalent cation tolerant protein CUTA, isoform CRA_c	A0A0G2JT00	Cuta	ML-dDG
Dedicator of cytokinesis 10	A0A0G2K0X2	Dock10	GL-dDG/GL-vDG
Dusp3 protein	B5DFF7	Dusp3	GL-dDG/GL-vDG
Eukaryotic translation initiation factor 5	Q07205	Eif5	GL-dDG/GL-vDG
Endoplasmic reticulum resident protein 29	P52555	Erp29	GL-vDG
Fumarylacetoacetate hydrolase domain-containing protein 2	B2RYW9	Fahd2	GL-dDG
ARF GTPase-activating protein GIT1	Q9Z272	Git1	GL-vDG
Guanine nucleotide-binding protein G(I)/G(S)/G(T) subunit beta-2	P54313	Gnb2	ML-vDG
Hyaluronan and proteoglycan link protein 4	D3Z9H2	Hapln4	GL-vDG
Inositol monophosphatase 1	F1M978	Impa1	GL-vDG
Uncharacterised protein	D4A4D5	LOC498555	GL-dDG
Uncharacterised protein	D4A5L9	LOC679794	ML-vDG
Myosin regulatory light chain 12B	A0A0G2JSW0	Myl12b	ML-vDG
NSF attachment protein gamma	A0A0G2K350	Napg	GL-dDG
N-myc downstream regulated gene 2, isoform CRA_a	A0A0G2JSV0	Ndrg2	GL-vDG
NADH dehydrogenase (Ubiquinone) Fe-S protein 3 (Predicted)	D3ZG43	Ndufs3	GL-dDG/ML-vDG
Ethanolamine-phosphate cytidylyltransferase	O88637	Pcyt2	GL-dDG
Phospholipase D3	Q5FVH2	Pld3	GL-vDG
Protein phosphatase methylesterase 1	Q4FZT2	Ppme1	GL-vDG
PC4 and SFRS1-interacting protein	Q566D6	Psip1	GL-dDG/GL-vDG
Proteasome subunit alpha type-2	P17220	Psma2	GL-dDG
Ptges3 protein	B2GV92	Ptges3	GL-vDG
Zero beta-globin	Q63011	Q63011	ML-dDG/GL-vDG
RAN-binding protein 3	M0R5Q3	Ranbp3	GL-dDG/GL-vDG
RCG48018	Q6AYA7	Rfk	GL-dDG
40 S ribosomal protein S8	B2RYR8	Rps8	GL-vDG
RuvB-like 1	P60123	Ruvbl1	GL-dDG/GL-vDG
SAP domain-containing ribonucleoprotein	Q498U4	Sarnp	GL-dDG
Clathrin coat assembly protein AP180	A0A0G2K0B6	Snap91	ML-dDG
LOC683667 protein	B0BNJ1	Sri	GL-dDG
RCG61099, isoform CRA_b	A0A0U1RRV7	Srsf3	GL-vDG
Thyroid hormone receptor-associated protein 3	Q5M7V8	Thrap3	GL-dDG
Target of myb1-like 2 membrane-trafficking protein	A0A0G2K9L2	Tom1l2	GL-vDG
Tubulin polymerisation-promoting protein family member 3	Q5PPN5	Tppp3	ML-dDG/ML-vDG
Cytochrome b-c1 complex subunit 2, mitochondrial	P32551	Uqcrc2	ML-vDG
Cytochrome b-c1 complex subunit 8	Q7TQ16	Uqcrq	GL-dDG/GL-vDG
ZW10 interactor	Q8VIL3	Zwint	GL-dDG

Notes: GL-dDG: granule cell layer of the dorsal dentate gyrus; ML-dDG: molecular layer of the dorsal dentate gyrus; GL-vDG: granule cell layer of the ventral dentate gyrus; ML-vDG: molecular layer of the ventral dentate gyrus.

**Figure 7:**
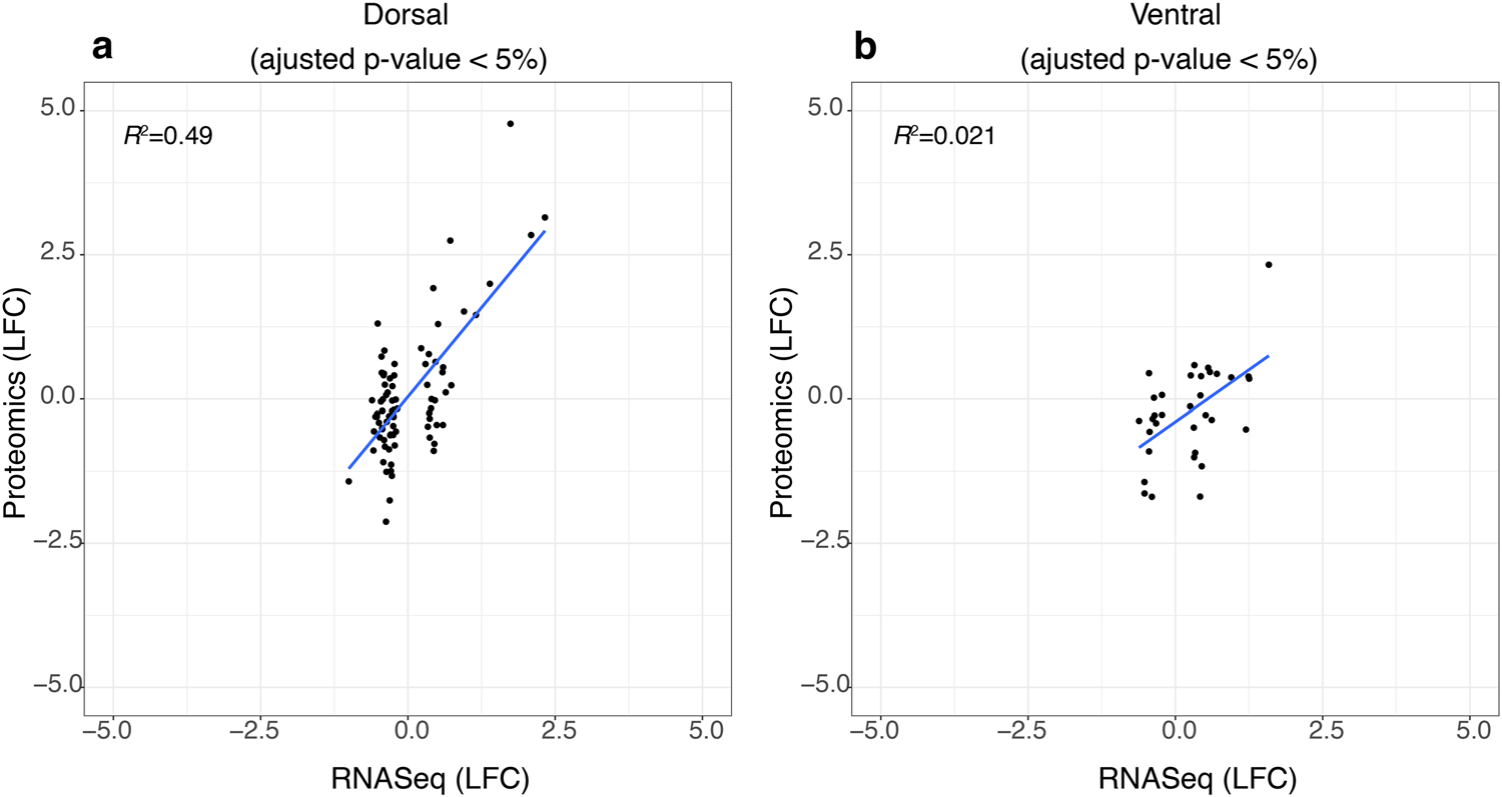
Correlation analysis between proteomics and RNA-sequencing data. The plots represent the correlation analysis between the proteomics data obtained in the current study and the RNA-sequencing data obtained through a previous study^24^. (**a**,**b**) Only the transcripts with adjusted p-values < 0.05 were included. The black dots represent the data values and the blue lines indicate the relationship between the LFC (log-fold change) of the transcripts in both platforms.

We also performed RNA-sequencing in the tissue obtained from the same animals used for proteomics analysis^24^ (Supplementary Dataset 13). In Table 2, we show the proteins and their respective transcripts identified in both of these studies^24^. To further explore our findings, we implemented a correlation analysis between the proteomics data obtained in the current study and the RNA-sequencing data acquired in the previous work^24^. Overall, our results showed a weak linear association between the data from both platforms, achieving R^2^ statistics of 0.03 and 0.003 for dorsal and ventral regions, respectively. However, when we selected only the significant transcripts identified as differentially expressed in the RNA-sequencing experiment, we found an improvement in the correlation with the proteomics data (Fig. 7), with R^2^ statistics of 0.49 and 0.02 for dorsal and ventral regions, respectively.

**Table 2:** Transcripts and proteins identified by the two OMIC approaches, transcriptomics^24^ and the proteomics results reported in the present work. Proteins marked with an asterisk (*) are further discussed in the text.

Gene symbol	Transcript ID	Protein ID	Log fold change RNA	Log fold change protein	Region
Cap2	ENSRNOG00000043350	P52481	−0.407	−1.44	vDG
Cpne7	ENSRNOG00000015397	D3ZWR4	−0.73	−0.7	vDG
Cryl1	ENSRNOG00000008989	Q811X6	0.49	0.86	dDG
Cst3*	ENSRNOG00000005195	P14841	1.12	1.32	dDG
Gap43*	ENSRNOG00000023433	P07936	15.44	−1.66	vDG
Gda	ENSRNOG00000018282	Q9JKB7	−0.22	−0.85	dDG
Gfap*	ENSRNOG00000002919	A0A1W2Q658	4.13	1.38	dDG
Gfap*	ENSRNOG00000002919	A0A1W2Q658	2.37	2.33	vDG
Glul	ENSRNOG00000049560	P09606	0.3	−1.23	dDG
Me3	ENSRNOG00000017311	A0A0G2K4C6	0.32	−1.16	vDG
Napg	ENSRNOG00000018914	A0A0G2K350	−0.194	−1.32	dDG
Pcyt2	ENSRNOG00000036684	O88637	−0.194	−1.26	dDG
Plpbp	ENSRNOG00000013751	D3ZCA0	−0.156	0.79	vDG
Ppp3r1	ENSRNOG00000043210	A0A0H2UHV6	−0.3	6.82	vDG
Sept7	ENSRNOG00000006545	F1LMC7	−0.121	−0.9	vDG
Sept7	ENSRNOG00000006545	F1LMC7	−0.12	−1.21	vDG
Snapq1	ENSRNOG00000023861	A0A0G2K0B6	0.157	−1.53	dDG
Sri	ENSRNOG00000049780	BOBNJ1	−0.242	−1.76	dDG
Syngr3	ENSRNOG00000012620	D4ABK1	−0.2	1.35	vDG
Tuba4a*	ENSRNOG00000003597	Q5XIF6	−0.31	−1.48	vDG
Vim*	ENSRNOG00000018087	G3V8C3	4.88	1.5	dDG
Wasf1	ENSRNOG00000047476	Q5BJU7	−0.28	−0.82	vDG

Notes: dDG: dorsal dentate gyrus; vDG: ventral dentate gyrus.

## Discussion

The use of animal models is essential for understanding the pathophysiology and development of chronic disorders such as MTLE. The perforant pathway stimulation, which was performed for eight hours, leads to non-detectable phenotypic manifestations. Therefore, its key feature is that it does not present a clinical recognisable *status epilepticus*, but it shows the development of spontaneous seizures after a latent period of approximately 21 days. These animals with spontaneous seizures display a pattern of neuronal damage that is similar to the HS lesion present in patients with MTLE^22,24^. In this study, we analysed the animals 15 days after electrical stimulation, a period in which the molecular changes induced by the electric stimulation have already taken place. However, the animals still did not present spontaneous seizures^24,26^. Changes in protein expression induced by acute seizures may differ from those related to the underlying epileptogenesis^27,28^. Therefore, the time point chosen was sufficient to reflect only the molecular mechanisms involved in the epileptogenesis process in the PPS model, without the risk of identifying pathways related mainly to acute seizures.

Proteins are the executive molecules of the cell, and they regulate biological processes. Therefore, proteomics allows us to better understand complex mechanisms by determining protein abundance patterns in a disease state. Here, for the first time, we report the differentially abundant proteins from the two layers (granule cell and molecular) in the two sub-regions (dorsal and ventral) of the hippocampus DG in animals submitted to the PPS protocol. The granule cell layer is mainly composed of granule cells, which is the only type of cell that gives rise to axons that innervate other hippocampal areas. There is also the mossy cell, whose axons leave the DG from one side to innervate the contralateral DG. The granule cell layer also contains numerous types of interneurons, mainly inhibitory. The molecular layer is primarily composed of dendrites of the granule cells, as well as axons from the enthorrinal cortex and other sources^29^. However, the types of cells are the same for each layer in each subfield, dorsal and ventral DG. Nonetheless, the different connections that each subfield receives, and the differential gene expression patterns seen in the subfield, as described by Fanselow and Dong^30^, do change, leading to different functions.

An important strength of the present work is the availability of previously published RNA-sequencing data^24^. Therefore, to make the comparison of the two genomic strategies meaningful, we chose to use the same tissue samples as in the previous study, thus eliminating biological variability and taking advantage of the RNA-sequencing data as a validation strategy for the current work. However, we acknowledge that by using a spatially defined cell population of interest, we are not able to apply more traditional validation techniques, such as western blot, due to the limited amount of protein obtained. Nonetheless, we are confident in the biological validity of the results presented here since the technique we used, mass spectrometry, is highly sensitive and accurate. Furthermore, consistent with our previous report on the transcriptome of the PPS animal model^24^, we identified a different profile of protein expression in the dorsal and ventral portions of the DG in the PPS model of MTLE.

Yet, we found two important pathways present in all layers and regions of the DG: inflammation and energy metabolism. These two pathways have been linked to many neurological disorders, and this has also been found in our transcriptomics results^24^. Thus, inflammation-related pathways seem to be activated in the PPS model, and the most prevalent pathways and biological processes identified were related to immune response, suggesting either an active phagocytosis process or infiltration of immune system cells in the DG^24^. Different inflammatory pathways were present in both regions of the DG, as also reported in the transcriptomics study^24^. Considering the PPI analysis, our results also point to enrichment of inflammation-related pathways in the ventral DG. In adult rodents, the generation of recurrent seizures, either by chemoconvulsants or electrical stimulation, triggers the rapid induction of inflammatory mediators in brain regions related to seizure activity onset and propagation^26^. Therefore, inflammation may be a consequence, as well as a cause of epilepsy. Brain inflammation can occur in types of epilepsy that are not linked to immunological dysfunction, indicating that chronic inflammation might be linked to some epilepsies, independent of the presence of initial insults or causes^27,28^. Seizures can induce brain inflammation that may become chronic, leading to progressive cell loss and inducing additional seizures, which can contribute further to inflammation^28,31^. Indeed, the PPS model used in the present study presents severe neuronal loss due to the induction of epilepsy, indicating the occurrence of an active process of inflammation during epileptogenesis. Similarly to what we found in the PPS model, pilocarpine and kainic acid-induced epilepsy models, as well as tissue from patients with MTLE share common neuroinflammatory response in brain areas affected by the injury caused either by the presence of a precipitant insult (*Status epilepticus* in animals) or by recurrent seizures^26–31^. Furthermore, we found that some of the identified enriched inflammatory pathways (immune response-CRTH2 signalling in Th2 cells, immune response-MIF-the neuroendocrine-macrophage connector, immune response-function of MEF2 in T lymphocytes, immune response-IL-16 signalling pathway) had PKC (in the GL-vDG), cPKC (in the GL-vDG), and the 14-3-3 protein (in the GL-vDG) as the main proteins abnormally regulated. These are known to be involved in signal transduction, and PKC, in particular, is responsible for phosphorylating many classes of proteins inside the cells (UniProt database). Therefore, as these proteins are downregulated, we suggest that these changes may be due to a compensatory mechanism aiming to decrease the inflammatory processes during epileptogenesis in the PPS model.

The second major pathway enriched in both layers and regions of the DG was energy metabolism. This result was also seen in the PPI analysis, and the main enriched pathways were metabolism (dDG and vDG), the citric acid cycle (vDG), gluconeogenesis (vDG), and amino acid metabolism (vDG*)*. Remarkably, in all energy-related enriched pathways, we found downregulation of the identified proteins. Mitochondrial oxidative phosphorylation is the primary source of ATP in neurons, and patients with HS can have mitochondrial ultra-structural abnormalities in DG neurons^32^. The combination of mitochondrial dysfunction and high neuronal excitability leads to an intracellular decrease in ATP levels and abnormal calcium homeostasis^33^. Animals with epilepsy induced by kainic acid have deficits in mitochondrial function^34^. Oxidative phosphorylation is also responsible for maintaining cell membrane potential using the Na^++^/K^+^ pump ATPase^35^. Therefore, changes in energy metabolism can be directly linked to epileptogenesis.

We found two other proteins that were upregulated in PPS animals in both layers and regions of the DG: GFAP (in the GL-dDG and GL-vDG) and vimentin (in the GL-dDG). These changes indicate an active process of astrogliosis and tissue damage, probably induced by neuronal death resulting from the stimulation performed in PPS animals.

It is noteworthy that cystatin-C (Cys-C) was differentially expressed in a specific layer and region of the DG, and it was upregulated in the GL-dDG of the PPS rats. Cys-C has been found to be abnormally expressed in an animal model of chronic epilepsy induced by the stimulation of the amygdala^36^, and it has been implicated in neuronal repair as a response to injury caused by ischemia, seizures, and other types of damage to the nervous system^37^. Therefore, the upregulation of Cys-C in the PPS model is likely to represent a defence mechanism against neuronal injury. Furthermore, the enrichment of the cell adhesion-gap junction pathway in the ML-vDG was represented mainly by the dysregulation of the structural protein tubulin alpha, tubulin (in microtubules), and tubulin beta. We also found that the transcript of connexin 31 is downregulated exclusively in the ML-vDG. The tubulin proteins and connexin 31 are present in gap junctions, and connexin 31 is responsible for the electrical coupling of cells, having an essential role in the synchronisation of neuronal activity^38,39^. Therefore, changes in proteins related to gap junctions, mainly connexin 31, is a new molecular mechanism to be further investigated in epilepsy. In addition, the downregulation of proteins involved in electrical coupling could alter the ionic balance in the neurons.

Interestingly, we found that a given protein could be regulated in the opposite direction in different regions of the DG in PPS animals. RACK1 was upregulated in the GL-dDG and downregulated in the GL-vDG. RACK1 is associated with various cell signalling pathways, especially those involving the cell membrane^40,41^. RACK1 has been associated with synapse plasticity, neurogenesis, and neuronal migration, and its decreased expression is known to result in seizures and epilepsy^40,42^. Furthermore, RACK1 is a scaffolding protein that is responsible for activating PKC, which in turn is associated with some of the enriched pathways found in our study, besides inflammation, such as activity-dependent synaptic AMPA receptor removal, constitutive and regulated NMDA receptor trafficking and signal transduction (mTORC2 downstream signalling), in the GL-vDG. In addition, RACK1 could be involved in the regulation of *SCN1A*, a gene that encodes the sodium voltage-gated channel alpha subunit 1 protein. *SCN1A* mutations cause Dravet syndrome, an epileptic encephalopathy, and genetic epilepsy with febrile seizures plus (GEFS^+^)^43^.

We also identified that the PARK7 protein was downregulated in the GL-vDG and upregulated in the GL-dDG. PARK7 has been involved in some types of Parkinson´s disease and other neurological disorders^44–46^. Furthermore, in disease models where mTORC1 is overactive, PARK7 expression is also increased, suggesting that PARK7 regulates mTORC1^46^. In turn, mTORC1 activity can induce rapid and dramatic remodelling of the synaptic proteome by regulating the synthesis of specific proteins and by altering the local expression of synaptic proteins^46^. Proteins in the mTORC1 signalling pathway seem to control the synthesis of GAP-43, which was found to be downregulated in the GL-vDG of PPS animals^46^. All these changes, together with the enrichment of the mTORC2 downstream signalling pathway, suggest that abnormal expression of PARK7 and GAP-43 might be a result of the dysregulation of the mTOR pathway in the PPS model. Indeed, the mTOR pathway has been shown to be involved in many neurological disorders, such as autism spectrum disorders^47,48^, Alzheimer’s disease, tuberous sclerosis complex^46,49,50^, focal cortical dysplasia^51,52^ and, more recently, intractable epilepsy^49,53–58^. The mTORC1 and mTORC2 complexes are also upregulated in tissue from patients with MTLE^59^. Therefore, our findings strengthen the relevance of the mTOR pathway in the mechanisms underlying MTLE^60^.

One may expect a high correlation between the expression levels of RNA and a protein^61,62^. However, the correlation between transcripts and their protein products has been described as surprisingly low and very dependent on the tissue species analysed^63–65^. Indeed, this correlation has been reported to be modest for human and chimpanzee brain tissue (R^2^ = 0.03 and R^2^ = 0.04, respectively)^66,67^. Moreover, this correlation varies in different cell types in the brain^68,69^. The discrepancies between the abundances of specific transcripts and proteins may be caused by the complexity and specificity of the transcription and translation mechanisms and are likely to involve variations in degradation rates, molecular abundances, alternative splicing, the occurrence of post-translation modification, and, especially, differences between transcription and translation rates^67,68,70^. In addition, we also found heterogeneity in the degree of correlation between transcripts and proteins in the different areas examined in our study and this correlation was lower in the dorsal DG.

## Conclusion

In conclusion, we report the proteomic profile of an animal model of MTLE, displaying the typical histopathological features of HS, as seen in patients with MTLE. Altogether, our results indicate that there are differences in the proteomic profile of differentially regulated proteins between different layers (granular and molecular layers) and different regions of the DG (ventral and dorsal portions). However, we also identified two major signalling pathways that are enriched in all layers and regions examined: inflammation and energy metabolism, both of which have been previously implicated in the mechanisms leading to epilepsy. In addition, we identified new signalling pathways and proteins present in specific layers and regions of the DG, such as PARK7, connexin 31/gap junction differential regulation in the vDG, and RACK1, which has opposite differential regulation in the dDG and vDG. These findings can now be further explored in additional studies. Finally, our results highlight the utility of high-throughput OMICS approaches in the study of disorders with complex and intricate mechanisms, such as epilepsy, and the importance of obtaining tissue from more circumscribed anatomic areas to fully appreciate the large biological heterogeneity present in the central nervous system.

## Methods

### Animals, electrode placement surgery, and performant pathway stimulation (PPS)

The experimental protocol was approved by the University of Campinas (UNICAMP) animal research ethics committee, which evaluates experimental protocols according to currently accepted ethical practices and legislation regarding animal research in Brazil [Brazilian federal law 11.794 (10/08/2008)]. We used the same animals (N = 8 for the ML and N = 7 for the GL; in this case, 3 animals were stimulated and 4 were sham controls) and procedures described in a previously published study aimed at reporting the transcriptome profile of PPS animals^24^.

### Brain slices and laser-capture microdissection

The frozen tissue, with 60-μm thickness, was serially sectioned in a Leica cryostat at −15 °C, covering the whole hippocampus. The tissue was mounted in PEN membrane-coated slides (Life Technologies), and they were immediately stained with Cresyl violet and dehydrated with ethanol. Microdissection was performed with a Palm Zeiss System. Samples were then manually collected using a forceps in 1.5-mL Axygen microtubes under surgical microscopy, where one tissue section was separated for microproteomics and one for transcriptomics.

### Sample preparation

Tissue samples were homogenised with 50 μL of urea (8 M) and incubated for 1.5 h at room temperature. Samples were subsequently centrifuged at 2000 g for 5 minutes, and the supernatant was quantified using the Qubit Protein Assay kit (Life Technologies, Waltan, MA, US). We used 30 μg of protein for each sample, which was reduced with 5 mM DTT (1,4-dithiothreitol, Sigma-Aldrich, St. Louis, MO, US) at 56 °C for 25 minutes and alkylated with 14 mM iodoacetamide (Sigma-Aldrich, St. Louis, MO, US) at room temperature for 30 minutes in the dark. After the addition of 1 mM CaCl_2_ (final concentration), proteins were digested with 0.6 μg of trypsin (Promega, Madison, WI, US sequence grade) by incubation for 16 h at room temperature. The reaction was stopped by adding TFA (trifluoroacetic acid, Sigma-Aldrich, St. Louis, MO, US) to a final concentration of 0.4%. Samples were centrifuged at 2500 g for 10 minutes at room temperature, and proteins were desalted using SepPack columns (Waters, C18) with acetonitrile (MS-grade acetonitrile from Sigma-Aldrich, St. Louis, MO, US), dried in SpeedVac vacuum concentrator (Eppendorf), and stored at −20 °C.

### Mass spectrometry analysis

Samples were reconstituted in 67.5 µL of 0.1% formic acid. An aliquot of 4.5 μL was analysed on an ETD enabled Orbitrap Velos mass spectrometer (Thermo Fisher Scientific, Waltham, MA, US) connected to an EASY-nLC system (Proxeon Biosystem, West Palm Beach, FL, US) through a Proxeon nanoelectrospray ion source. Peptides were separated by a 2–90% acetonitrile gradient in 0.1% formic acid using a PicoFrit analytical column (20 cm × ID75 μm, 5-μm particle size, New Objective) at a flow rate of 300 nL min^−1^ for 85 minutes. The nanoelectrospray voltage was set to 2.2 kV, and the source temperature was 275 °C. All instrumental methods were set up in the data-dependent acquisition mode (DDA). Full scan MS spectra (300–1600 m/z) were acquired in the Orbitrap analyser after accumulation to a target value of 1 × 106. The resolution in the Orbitrap was set to r = 60.000, and the 20 most intense peptide ions with charge state ≥2 were sequentially isolated to a target value of 5.000 and fragmented in the linear ion trap using low-energy CID (normalised collision energy of 35%). The signal threshold for triggering an MS/MS event was set to 1.000 counts. Dynamic exclusion was enabled with an exclusion size list of 500, an exclusion duration of 60 s, and a repeat count of 1. An activation of q = 0.25 and an activation time of 10 ms were used^71^.

### Bioinformatics analysis/statistical analyses

We performed the statistical modelling and inference by fitting linear mixed models as implemented in the MSstats R/Bioconductor package^72^. We did not consider any cut-off for fold change values since we believed that biological significance could be lost when this was done. To obtain enriched pathways, gene ontology, and networks involving the identified proteins, we used Metacore® software (Thomson Reuters). Multiple comparison correction was performed through the false discovery rate (FDR) at a level of 0.05, but it did not result in any significant protein (p.adjust). Therefore, we considered statistically significant results for which p-values were <0.05^73^. The PPI analyses were performed using the STRING database, and for pathway enrichment, we considered the Reactome database inside the STRING^74^.

### Construction of the imaging representing the dentate gyrus

A picture of one hippocampus from one naïve rat was obtained in an optical microscope, and the image was processed using Adobe Photoshop to build the schematic representation of the DG subfields.

## Supplementary information

Legend for supplementary material.

Supplementary Dataset 1.

Supplementary Dataset 2.

Supplementary Dataset 3.

Supplementary Dataset 4.

Supplementary Dataset 5.

Supplementary Dataset 6.

Supplementary Dataset 7.

Supplementary Dataset 8.

Supplementary Dataset 9.

Supplementary Dataset 10.

Supplementary Dataset 11.

Supplementary Dataset 12.

Supplementary Dataset 13.
