# Supplementary material for: Laser microdissection-based microproteomics of the hippocampus of a rat epilepsy model reveals regional differences in protein abundances
Source: Sci Rep. 2020 Mar 10;10:4412. doi: 10.1038/s41598-020-61401-8 (PMC7064578; doi:10.1038/s41598-020-61401-8)
Supplement: Supplementary file 1 — Legend for supplementary material. [file 41598_2020_61401_MOESM1_ESM.pdf]

Laser microdissection-based microproteomics of the hippocampus of a rat epilepsy model reveals regional differences in protein abundances

Amanda M. do Canto, Andre S. Vieira, Alexandre de Matos, Benilton S. Carvalho, Barbara Henning, Braxton A. Norwood, Sebastian Bauer, Felix Rosenow, Rovilson Gilioli, Fernando Cendes and Iscia Lopes-Cendes

Supplementary Dataset 1. Differentially expressed proteins found in the granular layer of the dorsal dentate gyrus (GL-dDG). The table shows details about the proteins identified as differentially expressed in the GL-dDG from the hippocampus of the PPS animals. We record name of proteins, Log2FC, p-value, adj.pvalue, accession number, gene symbol and uniqueness (whether the protein is only found in one specific region analysed). Included as a separate Excel document.

Supplementary Dataset 2. Complete list of significant GO processes (p-value <0.05) found in the GL-dDG. These results were obtained using the Metacore® software (Thomson Reuters) as described in the main text. Included as a separate Excel document.

Supplementary Dataset 3. Complete list of significantly enriched pathways (p-value <0.05) found in the GL-dDG. These results were obtained using the Metacore® software (Thomson Reuters) as described in the main text. Included as a separate Excel document.

Supplementary Dataset 4. Differentially expressed proteins found in the molecular layer of dorsal dentate gyrus (ML-dDG). The table shows details about the proteins identified as differentially expressed in the ML-dDG from the hippocampus of the PPS animals. We record name of proteins, Log2FC, p-value, adj.pvalue, accession number, gene symbol and uniqueness (whether the protein is only found in one specific region analysed). Included as a separate Excel document.

Supplementary Dataset 5. Complete list of significant GO processes (p-value <0.05) found in the ML-dDG. These results were obtained using the Metacore® software (Thomson Reuters) as described in the main text. Included as a separate Excel document.

Supplementary Dataset 6. Complete list of significantly enriched pathways (p-value <0.05) found in the ML-dDG. These results were obtained using the Metacore® software

(Thomson Reuters) as described in the main text. Included as a separate Excel document.

Supplementary Dataset 7. Differentially expressed proteins found in the granular layer of ventral dentate gyrus (GL-vDG). The table shows details about the proteins identified as differentially expressed in the GL-vDG from the hippocampus of the PPS animals. We record name of proteins, Log2FC, p-value, adj.pvalue, accession number, gene symbol and uniqueness (whether the protein is only found in one specific region analysed). Included as a separate Excel document.

Supplementary Dataset 8. Complete list of significant GO processes p-value <0.05) found in the GL-vDG. These results were obtained using the Metacore® software (Thomson Reuters) as described in the main text. Included as a separate Excel document.

Supplementary Dataset 9. Complete list of significantly enriched pathways (p-value <0.05) found in the GL-vDG. These results were obtained using the Metacore® software (Thomson Reuters) as described in the main text. Included as a separate Excel document.

Supplementary Dataset 10. Differentially expressed proteins found in the molecular layer of ventral dentate gyrus (ML-vDG). The table shows details about the proteins identified as differentially expressed in the ML-vDG from the hippocampus of the PPS animals. We record name of proteins, Log2FC, p-value, adj.pvalue, accession number, gene symbol and uniqueness (whether the protein is only found in one specific region analysed). Included as a separate Excel document.

Supplementary Dataset 11. Complete list of significant GO processes (p-value <0.05) found in the ML-dDG. The data These results were obtained using the Metacore® software (Thomson Reuters) as described in the main text. Included as a separate Excel document.

Supplementary Dataset 12. Complete list of significantly enriched pathways (p-value <0.05) found in the ML-dDG. These results were obtained using the Metacore® software (Thomson Reuters) as described in the main text. Included as a separate Excel document.

Supplementary Dataset 13. List of the top 10 proteins -up and downregulated, which do not match with any transcript identified in the transcriptomic data (data published in reference 24). Included as a separate Excel document.
